# Persistence and recovery of reproductive underperformance across successive parities in commercial sows

**DOI:** 10.3389/fvets.2026.1980366

**Published:** 2026-10-05

**Authors:** Nandipha Lili, Ziyanda Mpetile, Mthunzi Mndela, Oluwakamisi Festus Akinmoladun

**Affiliations:** 1 Department of Animal and Pasture Science, Faculty of Science and Agriculture, University of Fort Hare, Alice, Eastern Cape, South Africa; 2 Division of Agriculture and Food Technology, School of Agriculture, Geography, Environment, Ocean and Natural Sciences, The University of South Pacific, Samoa Campus, Apia, Samoa

**Keywords:** parity, reproductive recovery, reproductive underperformance, sow longevity, sow retention

## Abstract

**Background:**

Reproductive underperformance is commonly used to inform sow retention and replacement decisions, yet a single poor reproductive cycle may not necessarily indicate persistent reproductive failure. This study evaluated longitudinal patterns of reproductive recovery following low-performing parities and determined whether recovery was associated with previous performance history and reproductive stage.

**Methods:**

Historical production data comprising 15,699 farrowing records from 4,498 sows in a commercial breeding herd between 2020 and 2026 were retrospectively analysed. Reproductive underperformance was operationally defined as ≤10 piglets weaned (PW) per litter, corresponding to the 25th percentile of the observed distribution, while recovery was defined as >10 PW at the immediately subsequent consecutive parity.

**Results:**

Among 2,948 low-performance episodes with an observed immediately subsequent consecutive parity, 62.4% recovered, whereas 37.6% remained below the threshold; recovery estimates were therefore conditional on observation to the subsequent consecutive parity. Recovery declined from 63.8% following a first observed low-performing parity to 58.0% after two consecutive observed low-performing parities and 46.9% after three or more. After adjustment for reproductive and temporal factors, two consecutive observed low-performing parities were associated with 27% lower odds of recovery (OR = 0.73, 95% CI: 0.58-0.91; *P* = 0.005), while three or more were associated with 45% lower odds (OR = 0.55, 95% CI: 0.36-0.85; *P* = 0.007), compared with a first observed low-performing parity. Adjusted recovery probabilities following repeated underperformance were 61.0%, 55.1%, and 41.3% during early (P1–P2), prime (P3–P5), and later (P6–P8) reproductive stages, respectively; however, the overall stage × performance-history interaction was not significant (*P* = 0.121), and stage-specific patterns were therefore considered exploratory.

**Discussion/recommendation:**

These findings indicate that a single observed low-performing parity is frequently transient, whereas repeated underperformance is associated with progressively reduced recovery. Longitudinal reproductive history may therefore provide more informative evidence for sow retention decisions than the most recent litter alone, although external validation across commercial herds is required.

## 1 Introduction

Efficient management of breeding females is fundamental to the biological and economic sustainability of commercial pig production. Although genetic selection has substantially increased sow prolificacy, greater litter size does not necessarily translate proportionately into more piglets weaned or greater lifetime reproductive output because prenatal losses, stillbirths and pre-weaning mortality continue to constrain reproductive efficiency (1, 2). The productive value of a sow, therefore, depends not only on the size of an individual litter but on her ability to maintain satisfactory reproductive performance over successive parities. Consequently, decisions concerning sow retention and replacement represent an important component of breeding-herd management because premature removal may shorten productive life, whereas retaining females with persistent reproductive underperformance may reduce herd efficiency (3, 4).

Reproductive performance, however, is inherently dynamic. Litter outcomes vary between successive parities as a consequence of physiological maturity, maternal condition, genetic background, environmental conditions and management factors. Sow reproductive performance typically improves after the first parity, reaches its highest levels during intermediate parities and subsequently declines as females advance in reproductive age (5, 6). Maternal genetic line, season and herd-level temporal factors may further contribute to variation in litter size and piglet survival (7–9). Consequently, an unfavorable litter outcome at a particular parity may represent either a transient reproductive setback from which the sow subsequently recovers or the beginning of more persistent underperformance. This distinction is biologically and managerially important because the two patterns should not necessarily carry the same implications for retention.

Most previous investigations of sow reproductive performance have focused on determinants of litter size, reproductive losses, longevity or removal from the breeding herd (10, 11). Such studies have established important associations between parity, reproductive performance, and sow longevity, but evaluating individual parities in isolation provides limited information about the persistence of reproductive underperformance within the same female. Similarly, although previous reproductive performance is commonly considered when making herd-management decisions, considerably less attention has been directed toward quantifying the probability that a sow experiencing an underperforming reproductive cycle will recover at her subsequent parity and whether this probability changes as underperformance is repeated across consecutive reproductive cycles. This longitudinal distinction is particularly relevant because a single poor litter may have limited prognostic value if most affected females subsequently return to satisfactory performance.

Repeated reproductive records provide an opportunity to distinguish such transient variation from persistent reproductive underperformance. Rather than asking only whether performance differs among parities or whether current litter performance predicts the immediate subsequent litter, a longitudinal approach can determine whether the history and persistence of underperformance provide additional information about the likelihood of subsequent reproductive recovery. This distinction may be especially relevant across different stages of productive life. A low-performing litter occurring during early reproductive development may have different implications from repeated underperformance during prime or later reproductive stages, when the opportunity for future productive recovery becomes increasingly important to retention decisions. Evidence that recovery probabilities vary with the persistence of previous underperformance could therefore provide a more biologically informed basis for interpreting routine herd records.

Commercial herd databases are particularly valuable for addressing this question because they contain repeated reproductive observations from the same females under actual production conditions. Longitudinal production records have previously been used to investigate sow reproductive performance, longevity and lifetime productivity (12–14). However, their value extends beyond estimating conventional breed or parity effects: sequential records can be reconstructed into reproductive trajectories that quantify whether underperformance resolves or persists across successive cycles. Such an approach shifts the interpretation of routine production data from retrospective description toward evidence that may support sow-level retention decisions.

Therefore, the present study used longitudinal commercial breeding-herd records to characterize reproductive recovery following an underperforming parity. Specifically, the study aimed to (i) quantify the proportion of low-performing reproductive cycles followed by recovery at the immediately subsequent parity; (ii) determine whether the likelihood of recovery decreases with repeated consecutive reproductive underperformance; (iii) evaluate whether recovery patterns differ across early, prime and later reproductive stages; and (iv) identify reproductive, genetic and temporal factors associated with subsequent recovery. We hypothesized that a substantial proportion of single low-performing parities would be followed by recovery, whereas repeated consecutive underperformance would be associated with a progressively lower probability of recovery, with this pattern potentially becoming more pronounced during later reproductive life.

## 2 Materials and methods

### 2.1 Study site and herd management

This retrospective longitudinal study utilized historical production records from a large commercial farrow-to-finish pig breeding enterprise located in the Eastern Cape Province, South Africa. The farm operated an intensive production system comprising breeding, gestation, farrowing, nursery, grower, and finisher units under standardized husbandry practices. Records generated between January 2020 and June 2026 were included in the study. Ethical approval was obtained from the Animal Research Ethics Committee of the University of Fort Hare (Ref. no. AREC-2026-03-12-0002).

The breeding herd comprised two maternal genetic lines, Camborough and PIC Line, maintained under a routine artificial insemination programme. Pregnant sows were housed in designated gestation facilities and transferred to environmentally controlled farrowing units approximately one week before the expected farrowing date. Following parturition, sows remained with their litters until weaning, after which piglets were transferred to nursery facilities in accordance with standard farm procedures. Sows received nutritionally balanced diets formulated according to physiological stage, with drinking water available *ad libitum*. Routine vaccination, parasite control, biosecurity, and veterinary health-management programmes were implemented throughout the study period.

Reproductive events were routinely recorded by trained farm personnel using PigCHAMP^®^ herd-management software, enabling individual females to be followed across successive reproductive cycles.

### 2.2 Study design and data source

All available farrowing records within the study period were initially retrieved from the herd database. Records were screened for completeness, duplication, chronological consistency, and biological plausibility. Records lacking essential information, including animal identification, parity, or major litter-performance variables, were excluded. Following data cleaning, 15,699 farrowing records from 4,498 individual sows were retained.

Each sow was identified by a unique animal identification number (Animal_ID), allowing reproductive records to be ordered chronologically and linked across successive parities. The resulting dataset, therefore, had a longitudinal structure, with individual females contributing between one and ten farrowing records.

Internal consistency of the reproductive variables was also evaluated before longitudinal transition analyses. Total piglets born was verified against its component outcomes, while the relationships among piglets born alive, piglets available before weaning, piglet deaths, and piglets weaned were examined to identify biologically inconsistent records.

### 2.3 Reproductive variables

For each farrowing event, variables retrieved from the production database included total piglets born (TB), piglets born alive (BA), stillborn piglets (SB), mummified fetuses (MM), gestation length (GL; days), piglets available before weaning (PAW), piglets weaned (PW), and lactation length (LL; days).

Two reproductive-loss variables were derived from the primary records. Prenatal loss (PL) was calculated as


PL = SB + MM

and represented recorded fetal losses expressed as stillborn and mummified piglets. Piglet deaths during lactation (PD) were calculated as:


PD = PAW - PW

and represented piglet losses occurring between the number recorded as available before weaning and the number ultimately weaned.

Piglets weaned was used as the principal indicator of realized reproductive performance in the longitudinal recovery analysis because it represents the productive outcome achieved at completion of the farrowing-to-weaning cycle. Individual-level records of cross-fostering or piglet transfers, nurse-sow use, and the numbers of piglets fostered into or out of individual litters were not available in a form that could be reliably incorporated into the analysis. Consequently, PW was treated as a measure of realized weaning performance under the prevailing commercial herd-management conditions

### 2.4 Data screening and quality control

Records were screened for completeness, biological plausibility and logical consistency among related reproductive variables. The final analytical dataset contained complete observations for the principal reproductive variables, and no negative values for total born (TB), born alive (BA), stillborn (SB), mummified piglets (MM) or piglets weaned (PW). Internal consistency was verified by confirming that BA + SB + MM equalled TB and that prenatal loss equalled SB + MM. In addition, BA, SB and MM did not exceed TB, while PW did not exceed BA or the number of piglets available before weaning. The observed ranges in the final dataset were parity 1–10, TB 0–28, BA 0–25, SB 0–9, MM 0–6, piglets available before weaning 0–25, PW 0–22, gestation length 108–125 days and lactation length 14–41 days. These values represent the observed ranges of the retained analytical records rather than prespecified numerical exclusion thresholds. Individual-level information on cross-fostering, piglet transfers, fostering-in/out, and nurse-sow use was not available in the analytical dataset. Consequently, these management practices could not be explicitly identified or adjusted for during data screening or subsequent analyses.

### 2.5 Classification of parity and temporal variables

For the initial characterization of reproductive performance across the productive life of the sow, parity was classified as parity 1, 2, 3, 4, 5, 6, 7, and ≥8. The oldest parities were combined due to progressively smaller numbers of observations, while preserving the general biological progression of reproductive performance.

Farrowing season was determined from the farrowing date and classified according to Southern Hemisphere meteorological seasons as summer (December–February), autumn (March–May), winter (June–August), or spring (September–November). The farrowing year was treated as a categorical variable (2020–2026) to account for temporal variation in herd management, environmental conditions, and other unmeasured year-specific influences.

### 2.6 Definition of reproductive underperformance

The principal longitudinal analysis focused on whether reproductive underperformance represented a transient event followed by recovery or persisted across successive reproductive cycles. Piglets weaned was selected for defining underperformance because it represents the realized number of piglets successfully reaching weaning.

Reproductive underperformance was operationally defined as ≤10 piglets weaned per litter, corresponding to the 25th percentile of the PW distribution in the complete cleaned longitudinal dataset. The 25th percentile was also 10 piglets in the subset of records contributing to the parity-transition analysis, indicating consistency of the threshold across the analytical datasets. Because PW is a discrete count variable and multiple observations occurred at the threshold value, the proportion of observations classified as underperforming was not expected to correspond exactly to 25%.

The threshold was used as an empirical within-herd criterion for identifying comparatively low reproductive performance and was not intended to represent a universal commercial or biological threshold applicable across production systems.

### 2.7 Construction of consecutive parity transitions

To evaluate reproductive change within individual females, records were ordered by Animal_ID, parity, and farrowing date, and each reproductive record was linked to the subsequent record of the same sow. A transition was considered consecutive only when the subsequent recorded parity was exactly one parity greater than the current parity. Records involving gaps of more than one parity were excluded from the principal transition analysis because they could not reliably represent performance in the immediately subsequent reproductive cycle.

After applying this criterion, 11,172 consecutive parity transitions from parities 1–8 were available for longitudinal evaluation. Parity 9-to-10 transitions were excluded from substantive analyses because only five such transitions were available.

For each eligible transition, current-parity reproductive characteristics were linked to PW at the immediately subsequent parity. Of the 11,172 subsequent reproductive cycles, 3,485 (31.2%) met the ≤10-PW definition of reproductive underperformance.

### 2.8 Reproductive history, persistence and recovery

For each sow, low-performing reproductive cycles were arranged chronologically to determine whether underperformance occurred as an isolated event or persisted over consecutive parities. A running measure of consecutive low-performance episodes was constructed from the longitudinal reproductive history preceding each subsequent-parity outcome.

For the principal recovery analysis, reproductive history was classified into three categories:

- first observed low-performing parity, representing the first occurrence of reproductive underperformance in the immediately preceding sequence;
- two consecutive observed low-performing parities, and
- three or more consecutive observed low-performing parities.

Recovery analysis was restricted to low-performing current-parity episodes for which an immediately subsequent consecutive parity record was available. This resulted in 2,948 eligible low-performance episodes. Of these, 2,407 represented a first observed low-performing parity, 445 represented two consecutive observed low-performing parities, and 96 represented three or more consecutive observed low-performing parities.

Reproductive recovery was defined as a transition from ≤10 PW at the current parity to >10 PW at the immediately subsequent parity. Conversely, persistent underperformance was defined as ≤10 PW at both the current and subsequent parity.

This definition allowed the analysis to distinguish transient reproductive underperformance followed by return above the lower-quartile threshold from underperformance that continued into the subsequent reproductive cycle.

### 2.9 Subsequent-parity observation following low performance

To evaluate potential selection bias arising from conditioning the recovery analysis on continued observation, an additional analysis was conducted on all low-performing episodes (PW ≤10) occurring at parities 1–8 before requiring a subsequent parity record. Each episode was classified according to its preceding consecutive low-performance history as a first low parity, two consecutive low parities, or three or more consecutive low parities. The outcome was whether an immediately subsequent consecutive parity record was observed.

Because the study observation period ended in June 2026, episodes occurring during 2026 had a limited opportunity to contribute to a subsequent parity record. Therefore, the analysis was consequently restricted to low-performing episodes occurring from 2020 through 2025.

Differences in subsequent-parity observation across performance-history categories were initially evaluated using Pearson's chi-square test. A multivariable logistic regression model was then fitted with subsequent consecutive parity observation (yes/no) as the dependent variable and performance-history category as the principal explanatory variable, adjusting for current parity, maternal genetic line, year and season. Robust standard errors clustered by sow were used to account for repeated episodes contributed by individual sows. Associations are reported as odds ratios (OR) with 95% confidence intervals (CI).

### 2.10 Reproductive-stage classification

To determine whether recovery patterns differed by stage of productive life, current parity was further grouped into three reproductive stages: early (P1–P2), prime (P3–P5), and later (P6–P8).

These categories were based on the observed parity-associated reproductive trajectory in the herd and were intended to distinguish the early reproductive period, intermediate parities associated with comparatively high productivity, and later reproductive life. Parity 9 was excluded because of the very small number of eligible subsequent-parity transitions.

For analyses examining the joint effects of reproductive stage and previous performance history, reproductive history was additionally collapsed into first observed low-performing parity vs. repeated low-performing parities (≥2 consecutive observed low-performing parities). This binary classification reduced the number of sparse stage-by-history combinations while retaining the biological distinction between an isolated episode and repeated reproductive underperformance.

### 2.11 Statistical analysis

All analyses were conducted using R statistical software (version 4.5.1; R Foundation for Statistical Computing, Vienna, Austria). Statistical significance was defined as *P* < 0.05. Effect estimates are reported with 95% confidence intervals (CI).

Descriptive statistics were used to characterize the reproductive dataset and longitudinal transitions. Continuous variables were summarized using means and standard deviations and, where informative, medians and interquartile ranges. Categorical variables were summarized as frequencies and percentages.

To characterize the general reproductive trajectory of the herd and provide a biological context for the reproductive-stage classification, reproductive performance across parity was evaluated using mixed-effects models that accounted for repeated farrowing observations within individual sows. Continuous reproductive traits were analyzed using linear mixed-effects models, with breed, parity group, season, and year included as fixed effects and sow identity fitted as a random intercept. For PW, sow-level repeatability was additionally quantified from the variance components of the linear mixed-effects model as the intraclass correlation coefficient (ICC), calculated as the sow-level variance divided by the sum of sow-level and residual variances. Both an unadjusted ICC from an intercept-only model and an adjusted ICC from the model including maternal genetic line, parity group, year, and season were estimated.

Count-based reproductive-loss traits were evaluated using generalized linear mixed-effects models with an appropriate count distribution and log link. Model dispersion and residual behavior were evaluated using simulation-based diagnostics, and alternative count distributions were considered when evidence of overdispersion was present. Estimated marginal means were obtained for significant fixed effects, with Tukey adjustment applied to relevant pairwise comparisons.

Recovery frequency was first examined descriptively according to reproductive history, current parity, reproductive stage, and maternal genetic line. The proportion recovering at the immediate subsequent parity was calculated as:


$$\begin{array}{l} Recovery\left(\%\right)=\frac{number\;recovering\;to>10\;PW\;at\;the\;subsequent\;parity}{number\;of\;eligible\;low-performing\;episodes}\;\\ \;x\;100\; \end{array}$$

Corresponding proportions remaining at ≤10 PW were reported as persistent underperformance.

The primary inferential analysis evaluated whether the likelihood of recovery differed according to the persistence of previous reproductive underperformance. A multivariable logistic regression model was fitted with recovery (1 = recovery to >10 PW; 0 = continued performance ≤10 PW) as the binary dependent variable.

The principal explanatory variable was reproductive history, classified as first observed low-performing parity, two consecutive observed low-performing parities, or three or more consecutive observed low-performing parities. The model additionally included current parity, breed, current-parity PW, prenatal loss, gestation length, lactation length, farrowing year, and season. Because individual sows could contribute to more than one eligible low-performance episode, cluster-robust standard errors at the sow level were used to account for within-sow dependence. Model coefficients were exponentiated and expressed as odds ratios (OR) with 95% CI.

A secondary analysis examined whether the relationship between reproductive history and subsequent recovery varied according to reproductive stage. For this analysis, reproductive history was classified as first observed low-performing parity or repeated low-performing parities (≥2 consecutive episodes), and reproductive stage was classified as early, prime, or later. Reproductive stages were defined descriptively from the parity-associated trajectory observed within the study herd, with P1–P2 representing the early reproductive period, P3–P5 the higher-performing prime period, and P6–P8 the later reproductive period characterized by declining performance. These groupings were therefore herd-specific and data-informed rather than prespecified universal biological classifications, and stage-based analyses were considered exploratory.

A multivariable logistic regression model containing a reproductive stage × performance-history interaction was fitted, with adjustment for breed, current PW, prenatal loss, gestation length, lactation length, year, and season. Cluster-robust standard errors were again calculated at the sow level. Logistic regression models were fitted using base R, sow-clustered robust covariance estimates were obtained using the sandwich package and coefficient tests using lmtest, while adjusted marginal probabilities and contrasts were estimated using emmeans.

The association between reproductive stage and performance history was further examined by fitting a logistic regression model containing a reproductive stage × performance-history interaction, while adjusting for maternal genetic line, current-parity piglets weaned, prenatal loss, gestation length, lactation length, farrowing year and season. Dependence arising from repeated observations within sows was accounted for using sow-level cluster-robust variance estimation. The overall interaction was evaluated using a joint Wald test based on the cluster-robust covariance matrix. Model-adjusted recovery probabilities and 95% confidence intervals were estimated for each combination of reproductive stage and performance history. Stage-specific contrasts comparing repeated underperformance with a first observed low-performing parity were subsequently estimated; these contrasts were considered exploratory and their *P*-values were reported as nominal and unadjusted.

To assess whether associations between performance history and subsequent recovery were sensitive to the empirical ≤10-PW definition of underperformance, the primary recovery analysis was repeated using alternative thresholds of ≤ 9 and ≤ 11 PW. For each threshold, low-performance runs and performance-history categories were reconstructed independently, and recovery was defined as PW above the corresponding threshold at the immediately subsequent consecutive parity. The same model specification and sow-clustered robust standard errors used in the primary recovery model were retained. Estimates from the alternative thresholds were compared with those obtained using the primary ≤10-PW definition.

Because the binary recovery outcome does not capture the magnitude of change between consecutive parities, a complementary analysis was conducted using subsequent-parity PW as a continuous outcome among the same low-performance episodes included in the primary recovery analysis. A Gaussian linear regression model was fitted with subsequent-parity PW as the dependent variable and performance-history category as the principal explanatory factor. Current-parity PW, parity, maternal genetic line, prenatal loss, gestation length, lactation length, year, and season were included as covariates, consistent with the primary recovery model. Robust standard errors clustered by sow were used to account for repeated eligible episodes contributed by individual females. In addition, change in PW between consecutive parities (subsequent PW – current PW) and the proportions of episodes showing numerical improvement, no change, or decline were summarized descriptively by performance-history category.

## 3 Results

### 3.1 Characteristics of the study population and reproductive performance

A total of 15,699 farrowing records from 4,498 individual sows, collected between 2020 and 2026, were retained after data cleaning and quality control (Table 1). Annual farrowing records were relatively consistent from 2020 to 2025, ranging from 2,418 to 2,500 records, whereas fewer records were available for 2026 because data collection covered only part of the year. Overall, 3,284 sows (73.0%) contributed two or more farrowing records, providing repeated observations suitable for longitudinal evaluation of reproductive performance across successive parities. Individual sows contributed between 1 and 10 farrowing records, with a median of three records per sow. A flowchart of the entire analytical pipeline is shown in Figure 1.

**Table 1:** Characteristics of the study population and distribution of farrowing records in a commercial breeding herd (2020–2026).

Characteristic	Category	*n*	%
Study population	Individual sows	4,498	–
	Total farrowing records	15,699	–
	Study period	January 2020–June 2026	–
	Mean farrowing records per sow	3.49	–
	Median farrowing records per sow	3	–
	Maximum farrowing records per sow	10	–
	Sows with repeated farrowing records	3,284	73.0
	Sows with a single farrowing record	1,214	27.0
Breed	Camborough	13,009	82.87
	PIC Line	2,690	17.13
Parity group	1	3,707	23.61
	2	2,847	18.13
	3	2,282	14.54
	4	1,883	11.99
	5	1,587	10.11
	6	1,357	8.64
	7	1,056	6.73
	≥8	980	6.24
Season	Summer	4,049	25.79
	Autumn	4,337	27.63
	Winter	3,674	23.40
	Spring	3,639	23.18
Years of farrowing	2020	2,418	15.40
	2021	2,453	15.63
	2022	2,423	15.43
	2023	2,423	15.43
	2024	2,434	15.50
	2025	2,500	15.92
	2026†	1,048	6.68

Data represent 15,699 farrowing records obtained from 4,498 individual sows maintained in a commercial breeding herd between January 2020 and June 2026. Percentages for breed, parity group, season, and year are based on the total number of farrowing records (*n* = 15,699). †Records for 2026 represent a partial year because data collection ended in June 2026.

**Figure 1:**
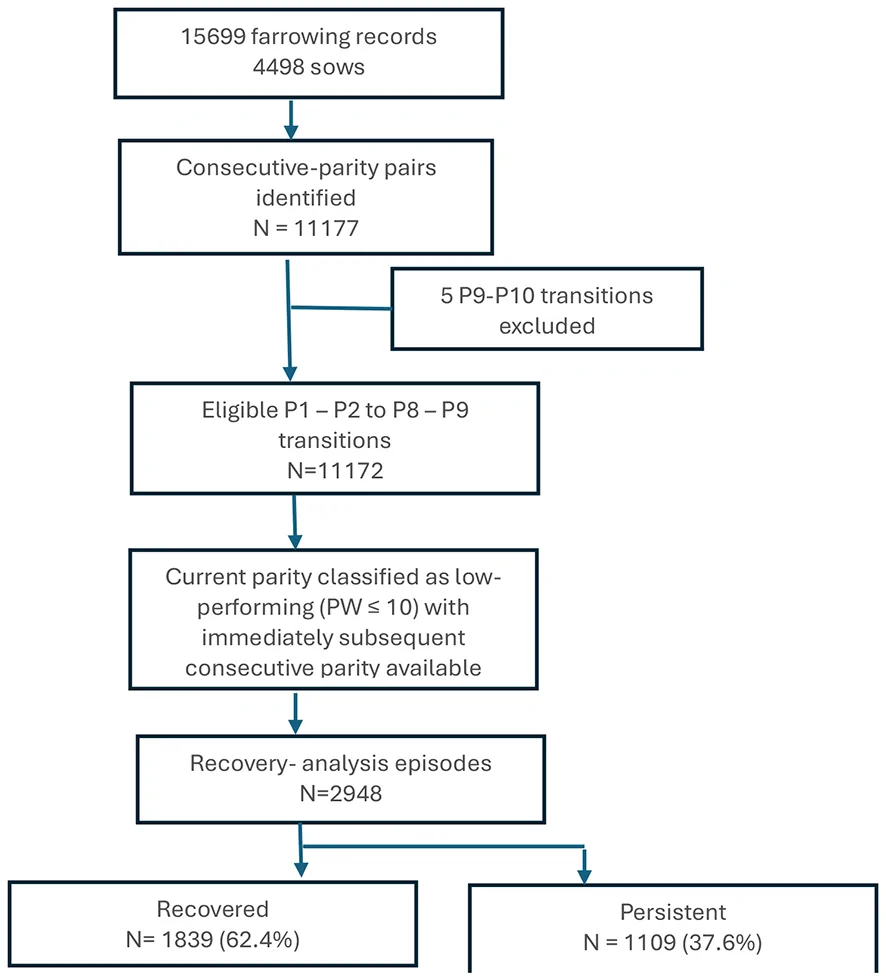
A flow chart for the entire analytical pipeline

The overall reproductive performance profile of the herd is presented in Table 2. Mean total litter size was 14.76 ± 3.22 piglets, of which 13.75 ± 3.10 were born alive. Recorded prenatal losses averaged 1.01 ± 1.16 piglets per litter, comprising 0.68 ± 0.89 stillborn piglets and 0.33 ± 0.64 mummified fetuses. An average of 12.73 ± 3.41 piglets were available before weaning, while 11.32 ± 2.98 piglets were weaned per litter, with a median of 12 piglets weaned. Gestation and lactation lengths averaged 115.72 ± 1.61 and 25.37 ± 2.64 days, respectively.

**Table 2:** Descriptive statistics of reproductive performance traits in a commercial breeding herd (*n* = 15,699 farrowing records).

Trait	*n*	Mean ±SD	Median	Minimum	Maximum	CV (%)
Total born	15,699	14.76 ± 3.22	15	0	28	21.8
Born alive	15,699	13.75 ± 3.10	14	0	25	22.6
Stillborn piglets	15,699	0.68 ± 0.89	0	0	9	130.2
Mummified fetuses	15,699	0.33 ± 0.64	0	0	6	194.9
Prenatal loss	15,699	1.01 ± 1.16	1	0	9	114.7
Piglets available before weaning	15,699	12.73 ± 3.41	13	0	25	26.7
Piglets weaned	15,699	11.32 ± 2.98	12	0	22	26.3
Gestation length (days)	15,699	115.72 ± 1.61	116	108	125	1.4
Lactation length (days)	15,699	25.37 ± 2.64	26	14	41	10.4

Importantly for the longitudinal recovery analysis, the 25th percentile of the piglets-weaned distribution was 10 piglets per litter. This value was therefore used as the operational threshold for identifying reproductive underperformance in subsequent parity-transition analyses. Thus, the descriptive population provided both the repeated within-sow records required for longitudinal evaluation and the empirical basis for distinguishing low-performing reproductive cycles from those above the lower-quartile threshold.

### 3.2 Parity-associated reproductive trajectory

Reproductive performance varied significantly across parity groups, with total born, born alive, piglets available before weaning, and piglets weaned all showing strong parity-associated differences (*P* < 0.001; Supplementary Table S1). Breed and year were also significantly associated with each of these traits (*P* < 0.001), whereas seasonal effects were comparatively small and inconsistent. Season was associated with born alive (*P* = 0.007), piglets available before weaning (*P* = 0.002), and piglets weaned (*P* = 0.002), but not total born (*P* = 0.080). Significant breed × parity interactions were observed for total born (*P* = 0.004) and born alive (*P* = 0.020), indicating that the magnitude of differences between the two maternal genetic lines was not constant across reproductive life.

The parity-specific trajectories of reproductive performance are illustrated in Figure 2. All four litter-performance traits followed a broadly similar pattern, with lower performance at first parity, improvement over the subsequent reproductive cycles, and the highest adjusted performance generally occurring during the middle parities.

**Figure 2:**
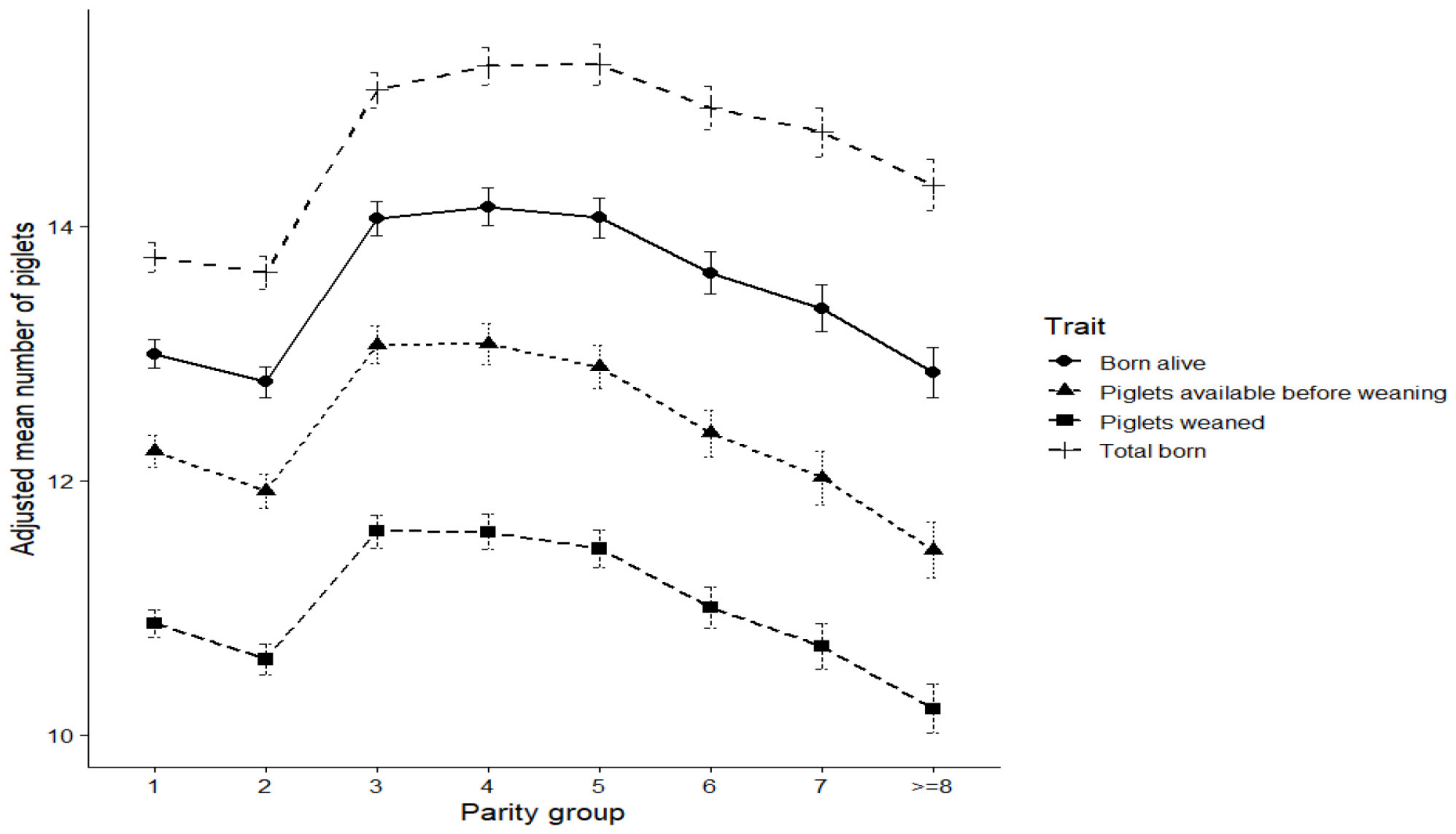
Integrated reproductive trajectories across successive parity groups in commercial sows. Adjusted means (± SE) are presented for total born (TB), born alive (BA), piglets available before weaning (PAW), and piglets weaned (PW).

Thereafter, reproductive output progressively declined toward the later parities. This pattern was particularly evident for weaned piglets, demonstrating that the probability of achieving a given level of litter output was assessed against a changing parity-associated performance background rather than a constant reproductive baseline.

Adjusted estimates further showed differences between the two maternal genetic lines, with Camborough sows generally having higher total born, born alive, piglets available before weaning, and piglets weaned than PIC Line sows (Supplementary Table S2). However, the significant breed × parity interactions for total born and born alive indicate that these breed differences should be interpreted in relation to parity rather than as uniform effects throughout reproductive life.

### 3.3 Construction and characteristics of consecutive reproductive transitions

To evaluate within-sow changes in reproductive performance across successive reproductive cycles, farrowing records were ordered chronologically within each sow and linked to the immediately subsequent recorded parity. Of the 4,498 sows represented in the complete dataset, 3,284 (73.0%) contributed at least two farrowing records. The number of records contributed per sow ranged from 1 to 10; 2,439 sows had at least three records, 1,853 had at least four, and 1,395 had five or more recorded reproductive cycles.

Across the 11,172 transitions retained for analysis, mean PW was 11.7 and 11.4 piglets at the current and subsequent parities, respectively (Table 3). The linear correspondence between PW in immediately consecutive parities was weak (r = 0.143), with parity-specific correlations ranging from 0.099 for P1 → P2 to 0.229 for P8 → P9. Complementary variance partitioning across all 15,699 farrowing records yielded an unadjusted sow-level ICC of 0.144. After adjustment for maternal genetic line, parity group, year, and season, the sow-level variance was 1.123 and the residual variance was 7.429, corresponding to an adjusted ICC of 0.131. Thus, approximately 13.1% of the residual variation in PW was attributable to persistent differences among sows after accounting for these systematic factors, indicating relatively low repeatability across reproductive cycles. Together, the weak consecutive-parity correlation and low adjusted ICC demonstrate substantial variation in reproductive output across cycles within individual sows, providing a basis for evaluating the occurrence, persistence, and subsequent recovery of reproductive underperformance.

**Table 3:** Distribution and piglets-weaned performance of consecutive parity transitions used to construct the longitudinal transition dataset.

Current parity	Subsequent parity	Consecutive transitions (*n*)	Mean (±SD) PW, current parity	Mean (±SD) PW, subsequent parity
1	2	2,675	11.4 ± 0.11	10.9 ± 0.07
2	3	2,103	11.2 ± 0.18	11.9 ± 0.18
3	4	1,742	12.1 ± 0.09	11.9 ± 0.18
4	5	1,464	12.1 ± 0.10	11.8 ± 0.17
5	6	1,240	12.0 ± 0.15	11.3 ± 0.12
6	7	989	11.8 ± 0.20	11.1 ± 0.11
7	8	681	11.8 ± 0.20	10.8 ± 0.06
8	9	278	11.9 ± 0.21	10.6 ± 0.04
Analytical dataset		11,172	11.7 ± 0.19	11.4 ± 0.13

PW, piglets weaned. Consecutive transitions were defined as records in which the subsequent recorded parity was exactly one parity greater than the current parity. Five P9 → P10 transitions were excluded from subsequent analyses because of the very small number of observations, leaving 11,172 transitions originating from parities 1–8. Twenty-four additional record pairs with a two-parity gap were excluded because the intervening reproductive cycle was not represented.

### 3.4 Recovery following reproductive underperformance

Among the 11,172 consecutive parity transitions retained for longitudinal analysis, reproductive underperformance was defined as ≤10 piglets weaned (PW) in the current parity, corresponding to the 25th percentile of the PW distribution. Because PW is a discrete count variable and multiple observations occurred at the threshold, the proportion classified as underperforming was not expected to correspond exactly to 25%. Restricting the recovery analysis to low-performing current-parity episodes for which an immediately subsequent consecutive parity was available yielded 2,948 eligible episodes. Of these, 1,839 (62.4%) recovered to >10 PW in the subsequent parity, whereas 1,109 (37.6%) remained underperforming ( ≤10 PW).

Recovery varied with the preceding pattern of reproductive underperformance (Figure 3). Among 2,407 episodes representing a first observed low-performing parity, 1,536 (63.8%) were followed by recovery in the subsequent reproductive cycle. Recovery declined to 58.0% (258/445) following two consecutive observed low-performing parities and to 46.9% (45/96) following three or more consecutive observed low-performing parities. Correspondingly, the proportion remaining underperforming increased from 36.2% after a first observed low-performing parity to 42.0% after two consecutive observed low-performing parities and 53.1% after three or more consecutive observed low-performing parities.

**Figure 3:**
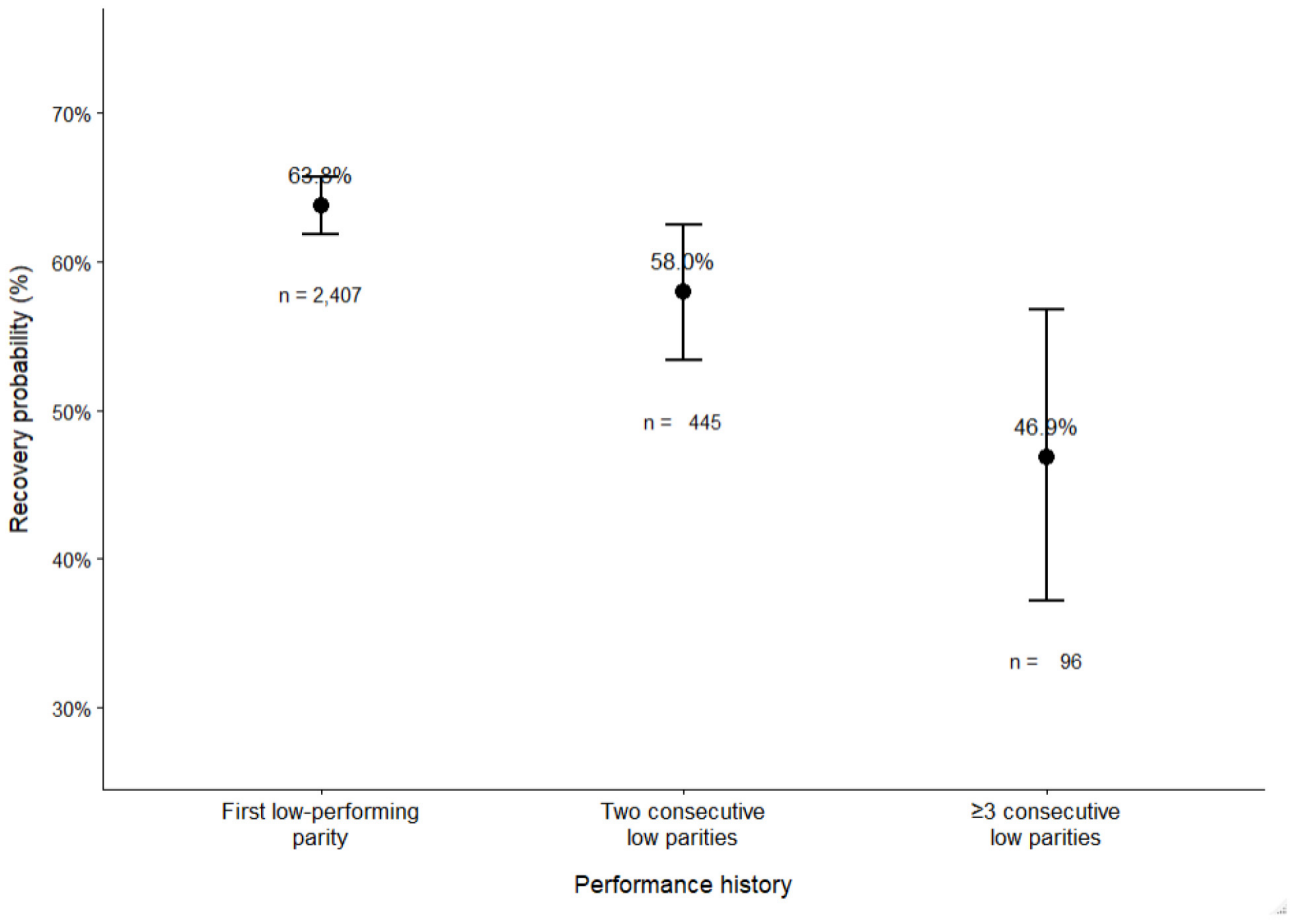
Recovery following reproductive underperformance according to preceding performance history. Points represent the observed proportion of low-performing episodes followed by recovery (>10 piglets weaned) in the immediately subsequent consecutive parity, and error bars represent 95% Wilson binomial confidence intervals. Reproductive underperformance was defined as ≤10 piglets weaned in the current parity. Numbers shown indicate the number of eligible low-performance episodes in each performance-history category.

The pattern of recovery also differed descriptively across reproductive stages and performance histories (Table 4). Recovery following a first observed low-performing parity was relatively frequent during the early and prime reproductive stages but was lower during the later reproductive stage. A similar decline across reproductive stages was more apparent among episodes of repeated underperformance, with the lowest recovery observed among repeatedly underperforming sows in the later reproductive stage. Because three or more consecutive low-performing parities cannot be accumulated within parities 1–2, this history category was not represented during the early reproductive stage. These descriptive patterns were subsequently evaluated using the adjusted reproductive stage × performance-history model.

**Table 4:** Recovery following reproductive underperformance according to reproductive stage and preceding performance history.

Reproductive stage	Performance history	Episodes (*n*)	Recovered (*n*)	Persistent (*n*)	Recovery (%)	Persistent (%)
Early (P1–P2)	First low parity	1,302	838	464	64.4	35.6
	Repeated low parities	163	104	59	63.8	36.2
Prime (P3–P5)	First low parity	736	485	251	65.9	34.1
	Repeated low parities	251	145	106	57.8	42.2
Later (P6–P8)	First low parity	369	213	156	57.7	42.3
	Repeated low parities	127	54	73	42.5	57.5
Total		2,948	1,839	62.4	1,109	37.6

Early, prime, and later reproductive stages were defined as current parities 1–2, 3–5, and 6–8, respectively. Recovery was defined as >10 piglets weaned in the immediately subsequent consecutive parity. No observations with three or more consecutive observed low-performing parities were possible within the early reproductive stage.

### 3.5 Subsequent-parity observation after low performance

Because recovery could only be evaluated when a subsequent consecutive parity was observed, the availability of follow-up after low performance was examined separately (Table 5). Among the 263 low-performing episodes occurring in 2026, 257 (97.7%) had no observed subsequent parity, reflecting substantial administrative right-censoring associated with the June 2026 study endpoint. The subsequent-parity analysis was therefore restricted to 4,597 low-performing episodes occurring during 2020–2025.

**Table 5:** Observation of an immediately subsequent consecutive parity following low reproductive performance according to performance history, 2020–2025.

Performance history	Low-performing episodes, *n*	Subsequent parity observed, *n* (%)	No subsequent parity observed, *n* (%)	Adjusted OR (95% CI)
First low parity	3,572	2,402 (67.2)	1,170 (32.8)	Reference
Two consecutive low parities	804	444 (55.2)	360 (44.8)	0.53 (0.45–0.63)
≥3 consecutive low parities	221	96 (43.4)	125 (56.6)	0.34 (0.24–0.47)
Overall	4,597	2,942 (64.0)	1,655 (36.0)	—

ORs were obtained from logistic regression models adjusted for current parity, maternal genetic line, year, and season, with robust standard errors clustered at the sow level. Episodes occurring in 2026 were excluded because of administrative right-censoring associated with the June 2026 study endpoint. Absence of a subsequent parity record should not be interpreted as confirmed culling or removal, because reasons for discontinuation were unavailable.

Two thousand nine hundred forty two of these episodes (64.0%) were followed by an observed immediately subsequent consecutive parity, whereas 1,655 (36.0%) were not. Subsequent-parity observation declined progressively with increasing persistence of low performance, from 67.2% (2,402/3,572) following a first low parity to 55.2% (444/804) following two consecutive low parities and 43.4% (96/221) following three or more consecutive low parities (χ^2^ = 83.75, df = 2, *P* < 0.001).

This association remained after adjustment for current parity, maternal genetic line, year and season. Compared with episodes representing a first low parity, the odds of an observed subsequent consecutive parity were lower after two consecutive low parities (OR = 0.53, 95% CI: 0.45–0.63, *P* < 0.001) and after three or more consecutive low parities (OR = 0.34, 95% CI: 0.24–0.47, *P* < 0.001). Thus, the declining probability of subsequent observation with increasing persistence of low performance was not attributable solely to differences in current parity.

### 3.6 Factors associated with subsequent reproductive recovery

Multivariable logistic regression was used to identify factors associated with recovery following reproductive underperformance, defined as a return to >10 piglets weaned in the immediately subsequent parity. Among the 2,948 low-performance episodes with an observed immediately subsequent consecutive parity, 1,839 (62.4%) recovered to >10 piglets weaned, whereas 1,109 (37.6%) remained at ≤10 piglets weaned. After adjustment for parity, breed, current piglets weaned, prenatal loss, gestation length, lactation length, farrowing year, and season, the persistence of preceding reproductive underperformance remained significantly associated with the probability of subsequent recovery (Figure 4). Relative to a first observed low-performing parity, the odds of recovery were 27.2% lower following two consecutive observed low-performing parities (OR = 0.728, 95% CI: 0.583–0.908; *P* = 0.005) and 44.7% lower following three or more consecutive observed low-performing parities (OR = 0.553, 95% CI: 0.360–0.850; *P* = 0.007). Thus, repeated low performance was associated with a progressively reduced likelihood of returning to above-threshold productivity in the subsequent reproductive cycle.

**Figure 4:**
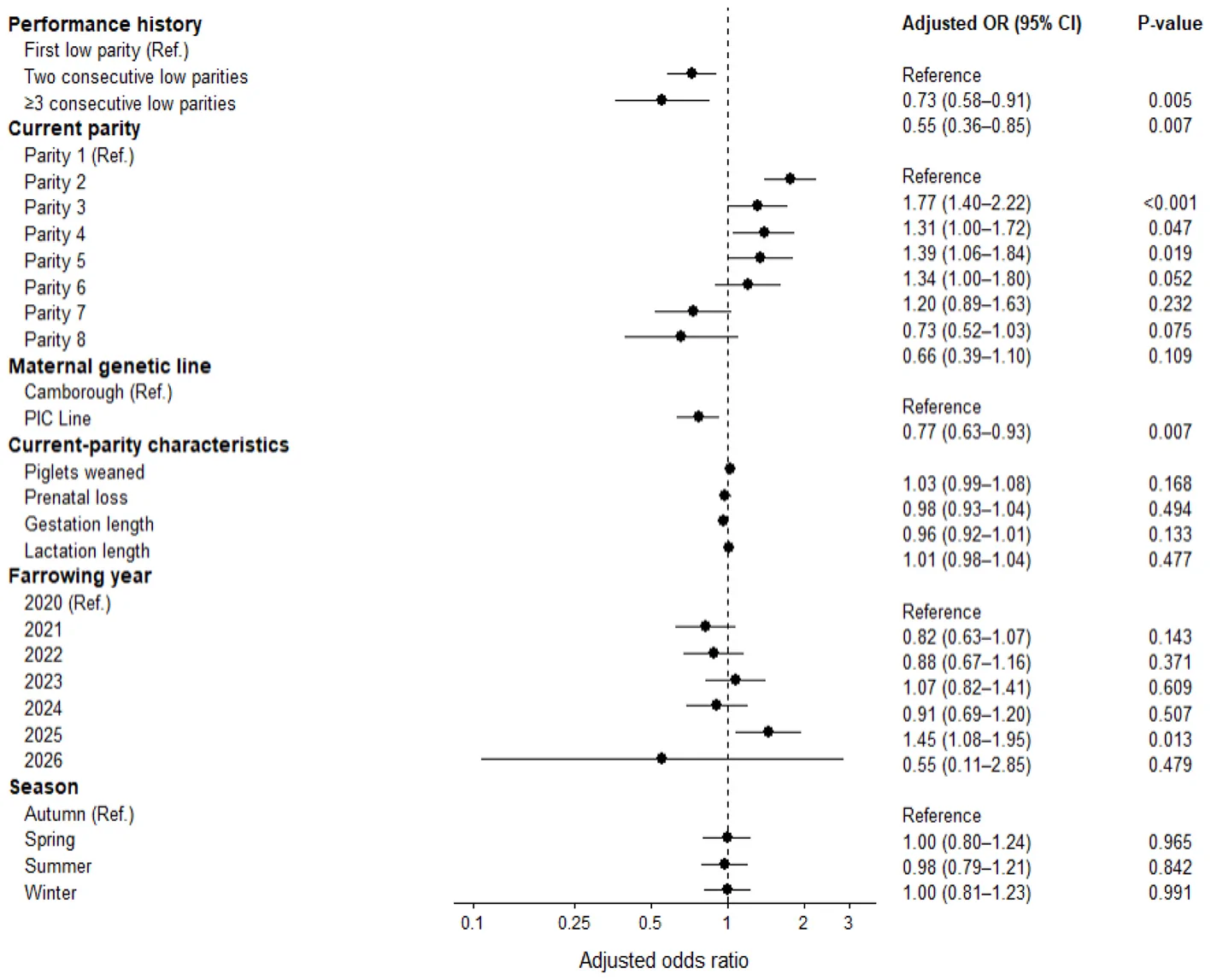
Factors associated with reproductive recovery following a low-performing parity. Points represent adjusted odds ratios and horizontal lines represent 95% confidence intervals from the multivariable logistic regression model. Recovery was defined as >10 piglets weaned in the immediately subsequent consecutive parity following reproductive underperformance (≤10 piglets weaned). Reference categories were first observed low-performing parity for performance history, parity 1 for current parity, Camborough for maternal genetic line, 2020 for farrowing year, and autumn for season. Odds ratios greater than 1 indicate higher odds of recovery, whereas values below 1 indicate lower odds of recovery.

Current parity was also associated with recovery. Compared with a low-performing episode at parity 1, recovery odds were higher at parity 2 (OR = 1.767, 95% CI: 1.403–2.225; *P* < 0.001), parity 3 (OR = 1.312, 95% CI: 1.004–1.716; *P* = 0.047), and parity 4 (OR = 1.392, 95% CI: 1.055–1.837; *P* = 0.019). The evidence for parity 5 was weaker (OR = 1.341, 95% CI: 0.998–1.801; *P* = 0.052), while recovery odds did not differ significantly from parity 1 at parities 6–8.

Maternal genetic line was independently associated with recovery. Compared with Camborough sows, PIC Line sows had 23.3% lower odds of recovering following a low-performing reproductive cycle (OR = 0.767, 95% CI: 0.633–0.929; *P* = 0.007). However, this breed-associated estimate should be interpreted cautiously because the two maternal genetic lines were unequally represented in the study population, with substantially fewer PIC Line sows available for comparison. In contrast, the number of piglets weaned within the low-performing current parity, prenatal loss, gestation length, lactation length, and season were not independently associated with subsequent recovery (*P* > 0.05). Among year effects, only 2025 differed significantly from the 2020 reference year, with greater odds of recovery (OR = 1.454, 95% CI: 1.083–1.953; *P* = 0.013). Cluster-robust standard errors accounting for repeated observations within individual sows produced essentially unchanged estimates and statistical inferences, supporting the robustness of the observed associations.

The association between persistence of underperformance and subsequent recovery was robust to alternative definitions of low performance. Using ≤ 9 PW, the adjusted odds of recovery were lower following two consecutive low parities (OR = 0.61, 95% CI: 0.43–0.86, *P* = 0.004) and ≥3 consecutive low parities (OR = 0.29, 95% CI: 0.13–0.65, *P* = 0.003) compared with a first low parity. Corresponding estimates using the primary ≤10-PW threshold were 0.73 (95% CI: 0.58–0.91, *P* = 0.005) and 0.55 (95% CI: 0.36–0.85, *P* = 0.007), respectively. At ≤ 11 PW, the corresponding ORs were 0.73 (95% CI: 0.62–0.86, *P* < 0.001) and 0.57 (95% CI: 0.44–0.74, *P* < 0.001). Although overall recovery decreased from 72.4% at ≤ 9 PW to 62.4% at ≤10 PW and 52.8% at ≤ 11 PW, the graded reduction in recovery odds with increasing persistence of underperformance was maintained across all three thresholds (Supplementary Table S3).

### 3.7 Magnitude of reproductive change following underperformance

Complementary analysis of PW as a continuous outcome supported the pattern observed with the binary recovery definition. Mean current-parity PW was similar among first low-parity episodes, two consecutive low-parity episodes and episodes following three or more consecutive low parities (8.37, 8.36 and 8.40 piglets, respectively). However, mean subsequent-parity PW declined across these groups from 11.00 to 10.70 and 9.65 piglets, respectively. Correspondingly, mean changes in PW were +2.64 ± 3.51, +2.32 ± 3.52 and +1.25 ± 3.62 piglets, with median changes of +3 (IQR 1–5), +2 (0–4) and +1 (−1–3), respectively. Numerical improvement occurred in 75.7%, 73.3% and 60.4% of episodes, whereas declines occurred in 16.5%, 19.1% and 30.2%, respectively.

After adjustment for current-parity PW and the other model covariates, subsequent-parity PW was 0.52 piglets lower following two consecutive low parities than following a first low parity (β = −0.52, 95% CI: −0.84 to −0.21, *P* = 0.001). Following three or more consecutive low parities, subsequent PW was 1.30 piglets lower (β = −1.30, 95% CI: −1.95 to −0.66, *P* < 0.001). Thus, increasing persistence of underperformance was associated not only with a lower probability of crossing the recovery threshold but also with a smaller magnitude of reproductive improvement and lower subsequent PW (Supplementary Table S4).

### 3.8 Recovery according to reproductive stage and performance history

Adjusted recovery probabilities differed descriptively according to reproductive stage and performance history (Figure 5). During the early reproductive stage (P1–P2), adjusted recovery probabilities were virtually identical between sows experiencing their first observed low-performing parity and those with repeated underperformance [60.9% (95% CI: 54.4–67.0%) vs. 61.0% (51.3–69.9%), respectively]. Greater separation was observed during the prime stage (P3–P5), where adjusted recovery probability was 62.3% (55.5–68.7%) following a first observed low-performing parity compared with 55.1% (46.4–63.4%) following repeated underperformance. During the later stage (P6–P8), the corresponding probabilities were 54.9% (46.9–62.7%) and 41.3% (31.3–52.1%), respectively.

**Figure 5:**
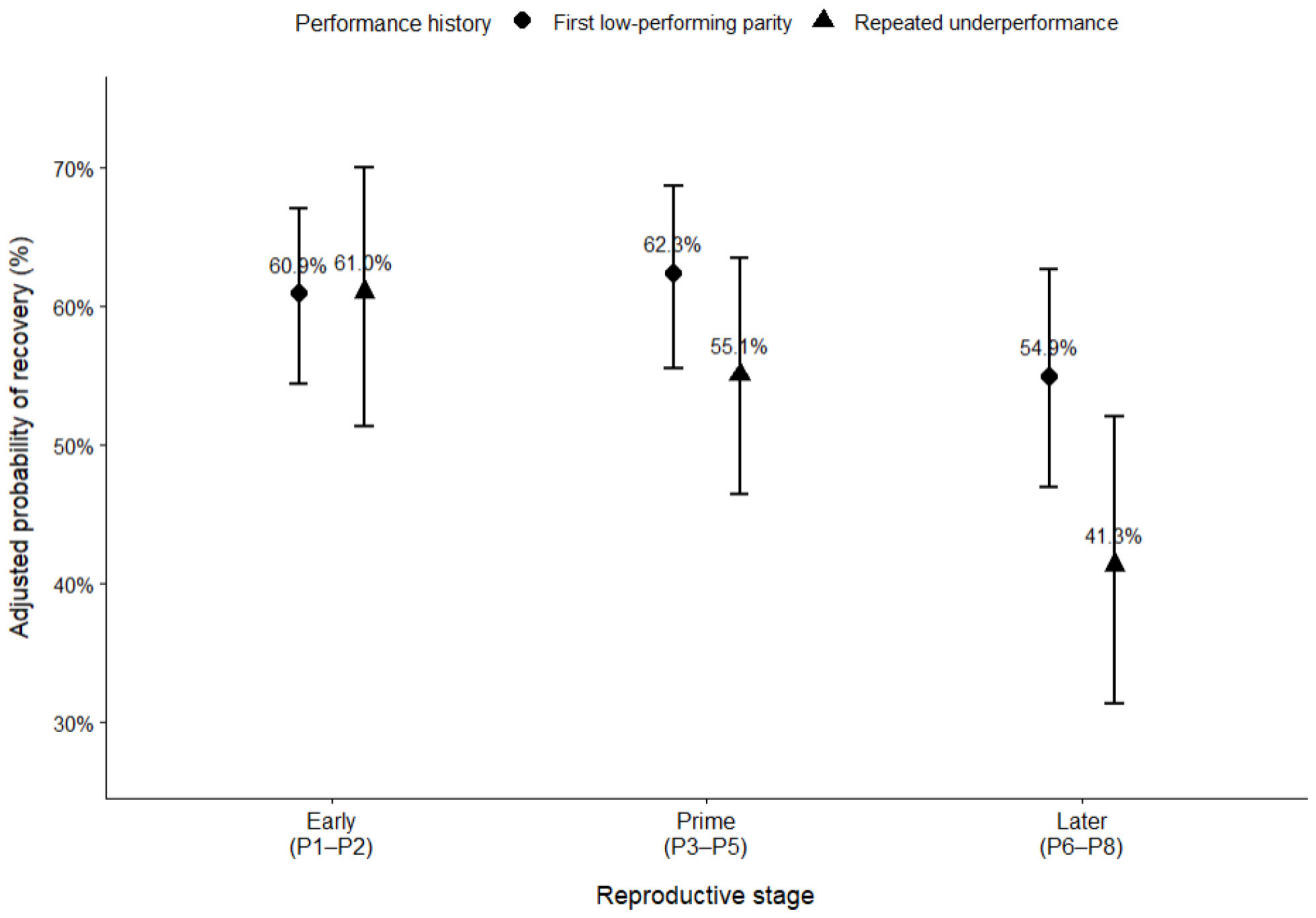
Adjusted probability of reproductive recovery according to reproductive stage and history of underperformance. Points represent model-adjusted probabilities of recovery, and error bars represent 95% confidence intervals based on sow-clustered robust variance estimates. Early, prime and later reproductive stages correspond to parities 1–2, 3–5 and 6–8, respectively. Recovery was defined as >10 piglets weaned in the immediately subsequent consecutive parity following reproductive underperformance ( ≤10 piglets weaned). The overall reproductive stage × performance-history interaction was not statistically significant (Wald χ^2^ = 4.22, df = 2, *P* = 0.121); stage-specific patterns should therefore be interpreted as exploratory.

In stage-specific contrasts (Table 6), repeated underperformance was not associated with recovery during the early stage (OR = 1.006, 95% CI: 0.714–1.418; nominal *P* = 0.974). Lower recovery odds were observed for repeated underperformance during the prime (OR = 0.741, 95% CI: 0.550–0.997; nominal *P* = 0.048) and later (OR = 0.578, 95% CI: 0.380–0.878; nominal *P* = 0.010) stages. However, the overall reproductive stage × performance-history interaction was not statistically significant (Wald χ^2^ = 4.22, df = 2, *P* = 0.121). Accordingly, the stage-specific contrasts were considered exploratory and should not be interpreted as evidence of a statistically established difference in the effect of performance history across reproductive stages.

**Table 6:** Stage-specific association between repeated reproductive underperformance and subsequent recovery.

Reproductive stage	Comparison	OR for recovery	95% CI	*nominal P*-value
Early (P1–P2)	Repeated vs first low parity	1.01	0.714–1.418	0.974
Prime (P3–P5)	Repeated vs first low parity	0.74	0.550–0.997	0.048
Later (P6–P8)	Repeated vs first low parity	0.58	0.380–0.878	0.010

OR, odds ratio; CI, confidence interval. Odds ratios compare repeated underperformance with a first observed low-performing parity within each reproductive stage. *P*-values are nominal, unadjusted stage-specific contrasts and should be interpreted as exploratory. The overall reproductive stage × performance-history interaction was not statistically significant (Wald χ^2^ = 4.22, df = 2, *P* = 0.121).

## 4 Discussion

The findings of this study shift the interpretation of reproductive underperformance from an isolated litter outcome to the longitudinal reproductive trajectory of the individual sow. This distinction is relevant to commercial herd management because inadequate reproductive performance contributes to planned sow removal, while longevity is increasingly recognized as an economically important component of breeding-herd efficiency (15, 16). Large-scale commercial studies have similarly demonstrated close relationships among reproductive performance, planned removal and sow longevity, highlighting the need to balance litter productivity with the duration of productive life (17, 18).

An important observation supporting this longitudinal perspective was the weak relationship between reproductive output in consecutive parities. Across 11,172 eligible parity transitions, the correlation between current- and subsequent-parity piglets weaned was only 0.143, with similarly weak correlations within individual parity classes. Thus, the numerical outcome of one litter provided relatively little information about the numerical outcome of the immediately succeeding litter. This is biologically plausible because the number of piglets ultimately weaned represents the integrated outcome of numerous processes, including ovulation and embryonic survival, maternal condition, litter size, farrowing outcome, lactational capacity, piglet viability, disease, nutrition and management (19–23). Several of these influences are transient and may therefore affect one reproductive cycle without necessarily recurring in the next. The documented effects of parity, body condition, housing and environmental conditions on sow reproductive performance further illustrate the considerable biological and management-related variation underlying individual litter outcomes (11, 24, 25).

This variability was reflected directly in the recovery analysis. Nearly two-thirds of first low-performing episodes were followed by immediate recovery above the threshold of ≤10 piglets weaned. Consequently, an isolated low-performing litter should not necessarily be interpreted as evidence of sustained reproductive failure. Sow reproductive performance is dynamic across productive life, with substantial within-sow and environmental variation across successive parities (26). The present study extends this principle by specifically examining the reproductive outcome immediately following an underperforming parity. From a management perspective, therefore, a single low-performing litter may be insufficient to distinguish a temporarily compromised sow from one developing persistent reproductive underperformance.

In contrast, the persistence of underperformance across successive parities was associated with a progressively lower probability of recovery. Importantly, this graded association was retained when underperformance was alternatively defined as ≤ 9 or ≤ 11 PW, indicating that the relationship between performance history and subsequent recovery was not dependent on the specific ≤10-PW cut-point. After adjustment for current parity and other measured covariates, sows with two consecutive observed low-performing parities had 27.2% lower odds of recovery than sows experiencing their first observed low-performing parity, while those with three or more consecutive observed low-performing parities had 44.7% lower recovery odds. This graded association is an important finding because it distinguishes between the occurrence of underperformance and its persistence. Repeated poor reproductive outcomes may increasingly reflect persistent biological or management-related constraints rather than temporary variation around a sow's underlying reproductive performance. Potential mechanisms include chronic body-condition problems, impaired maternal capacity, reproductive aging, locomotor or health disorders, persistent limitations in lactational performance, or other constraints not captured by routine herd records (27–29). These mechanisms were not directly measured in the present study and should therefore be regarded as plausible explanations rather than demonstrated causal pathways.

An important qualification to these recovery estimates is that recovery was observable only when a sow subsequently produced another consecutive parity record. In the complementary analysis restricted to low-performing episodes occurring during 2020–2025 to minimize end-of-study censoring, 67.2% of first low-parity episodes were followed by an observed consecutive parity, compared with 55.2% after two consecutive low parities and 43.4% after three or more consecutive low parities. This gradient remained after adjustment for current parity, maternal genetic line, year, and season, with the odds of an observed subsequent parity being 47% lower after two consecutive low parities and 66% lower after three or more consecutive low parities than after a first low parity. Thus, increasingly persistent underperformance was associated not only with a lower probability of recovery among subsequently observed sows, but also with a lower probability of contributing an observable subsequent consecutive parity. Consequently, the recovery estimates reported here are conditional on continued observation and should not be interpreted as unconditional recovery probabilities for all sows experiencing low performance.

Parity provided additional biological context for this recovery process. In the complete herd population, reproductive output increased from first parity, reached its highest levels during intermediate parities and subsequently declined in older sows. This trajectory aligns with the established pattern in commercial sow populations, in which reproductive performance improves as young females attain physiological maturity, then declines at advanced parities (11). These parity-related changes justify interpreting reproductive underperformance in terms of the sow's reproductive stage rather than applying the same biological expectation uniformly across all parities.

Consistent with this interpretation, adjusted odds of recovery following underperformance were greater at parities 2–4 than at parity 1, whereas no significant recovery advantage was evident at parities 6–8. Young sows are still partitioning nutrients among maternal growth, maintenance, pregnancy and lactation, whereas intermediate-parity females have reached greater physiological maturity and generally occupy the most productive stage of reproductive life. At advanced parities, accumulated physiological demands, reproductive aging and increasing health risks may reduce the capacity to return to satisfactory performance (23, 30). Evidence that mortality risk and previous reproductive characteristics vary with parity further supports the importance of reproductive stage when evaluating the future productive potential of a sow (31).

The combination of reproductive stage and performance history revealed an additional pattern of potential biological relevance. During early reproductive life, recovery following repeated underperformance was broadly comparable with that following a first observed low-performing parity. In contrast, recovery tended to be less favorable following repeated underperformance during the prime and, particularly, later reproductive stages. This pattern suggests that the persistence of poor performance may become more consequential as reproductive life advances. Older sows may have less physiological capacity to compensate for recurring reproductive challenges due to reproductive aging, cumulative metabolic demands, and increasing susceptibility to health or functional limitations. Nevertheless, reproductive stage cannot be considered a confirmed modifier of the relationship between performance history and recovery because the overall interaction was not statistically significant. The stage-specific findings should therefore be regarded as exploratory and hypothesis-generating rather than evidence for stage-specific retention thresholds. Larger multi-herd studies with greater representation of repeatedly underperforming older sows are needed to determine whether reproductive stage meaningfully modifies the likelihood of subsequent recovery.

The maternal genetic line was also independently associated with recovery. PIC Line sows had approximately 23% lower adjusted odds of recovery following an underperforming reproductive cycle than Camborough sows. The complete-herd analysis similarly showed generally higher reproductive output among Camborough females, although breed × parity interactions for total born and born alive indicated that genetic-line differences were not constant across reproductive life. Maternal genetic background can influence litter size, maternal capacity, piglet survival and longevity, and recent analyses of commercial maternal lines demonstrate that relationships among prolificacy, longevity and survival continue to change with genetic progress (32, 33).

Interestingly, several characteristics of the immediately preceding low-performing litter were not independently associated with recovery. Within litters already classified as low-performing, the actual number of piglets weaned, prenatal loss, gestation length and lactation length did not significantly distinguish sows that subsequently recovered from those that remained below the threshold. This does not imply that these traits are biologically unimportant. Rather, within the restricted range of low-performing litters, they provided little additional information after adjustment for the other model covariates. Important determinants unavailable in routine records, including body condition, feed intake, disease events, farrowing difficulty, locomotor health, teat functionality, cross-fostering and litter-management practices, could potentially explain additional variation in recovery and should be considered in future prospective studies.

Season likewise showed no independent association with recovery despite small seasonal differences in some reproductive traits in the complete herd. This suggests that, under the conditions of this intensive commercial enterprise, season contributed relatively little to determining whether an already underperforming sow recovered during the subsequent reproductive cycle. Environmental effects on reproduction remain strongly context-dependent; however, associations between climatic conditions and reproductive performance can vary among farms depending on housing, management, and thermal control systems (34, 35).

### 4.1 Implications for sow retention and reproductive management

The principal practical implication of this study is not that ≤10 piglets weaned should become a culling threshold. The threshold represented the lower quartile of reproductive performance within this particular herd and was intentionally defined as an empirical within-herd criterion rather than a universal biological or commercial benchmark. Instead, the findings demonstrate the potential value of incorporating performance history into the interpretation of reproductive records. Under such an approach, the first low-performing cycle would serve as a signal for closer evaluation rather than necessarily constituting sufficient evidence for immediate removal. Potential underlying causes, including sow condition, health, locomotion, lactational performance, farrowing complications and litter-management circumstances, could be considered alongside the reproductive record. Continued underperformance over subsequent consecutive parities would progressively strengthen the evidence that poor performance is persistent rather than transient.

This distinction may be important because both premature and delayed culling carry consequences. Removing a biologically sound sow following a temporary reproductive setback forfeits her remaining productive potential and requires the introduction of a replacement gilt, whereas retaining a sow with persistent reproductive limitations incurs feed, housing and opportunity costs while potentially reducing herd output. Contemporary work on sow longevity increasingly emphasizes this precise balance between productivity and retention. The present results suggest that longitudinal reproductive records can inform this decision by providing information not available from the most recent litter alone. In practical terms, therefore, the relevant question may not simply be “Did this sow underperform?,” but rather “Is this the first occurrence, or is it part of an emerging pattern of repeated underperformance?”

Importantly, the present analysis should not be interpreted as establishing an optimal culling rule. Recovery above the threshold is a biological outcome and not an economic decision criterion. A comprehensive retention model would additionally require replacement-gilt costs, feed and housing costs, expected remaining productive lifespan, non-productive days, health and welfare information, salvage value, genetic merit and the opportunity cost associated with retaining a lower-performing female. Nor does recovery to >10 piglets necessarily imply optimal performance. Rather, the study establishes an empirical foundation for a potentially more informative decision framework that considers reproductive history and stage alongside conventional current-parity measures. Future work could extend this approach by developing and externally validating sow-level prediction models that estimate the probability of future recovery and translate that probability into economically informed retention thresholds.

### 4.2 Strengths and limitations

A major strength of the present study is its longitudinal structure. The analysis included 15,699 farrowing records from 4,498 sows, with 73% of females contributing repeated observations. More importantly, the recovery analysis was based on genuinely consecutive reproductive records rather than simply comparing aggregate performance across parity groups. Reconstruction of 11,172 consecutive parity transitions enabled explicit linking of previous reproductive history to the immediately subsequent outcome in the same female. The use of sow-clustered robust standard errors further accounted for dependence that arises when individual females contribute multiple eligible low-performance episodes, and the stability of the estimates after robust adjustment supports the statistical reliability of the principal associations.

Several limitations nevertheless require consideration. First, the study was conducted in a single commercial herd; therefore, the ≤10-PW threshold, absolute recovery probabilities and genetic-line effects should not be assumed to generalize directly to herds with different genetics, management systems, disease status or productivity levels. Second, reproductive underperformance was operationally defined from piglets weaned. Although this is a commercially meaningful endpoint that integrates reproductive output and survival to weaning, it can also be influenced by litter-management interventions such as cross-fostering, nurse-sow practices, and piglet transfers. These management events were not available in sufficient detail to be incorporated into the present analysis. Third, the retrospective database lacked potentially important sow-level explanatory variables, including body condition and backfat, feed intake, health and treatment records, locomotor status, farrowing duration and assistance, teat and udder condition, disease events and reasons for eventual culling. Consequently, the identified associations should not be interpreted causally.

A further limitation concerns potential selection arising from the requirement for an observed subsequent consecutive parity. The complementary analysis demonstrated that subsequent-parity observation itself declined with increasing persistence of underperformance. However, absence of a subsequent parity record cannot be equated directly with culling because explicit dates and reasons for herd exit were unavailable. Disappearance from subsequent reproductive records could reflect culling, mortality, sale or transfer, reproductive failure, or administrative censoring. The latter was particularly important in 2026, when 97.7% of low-performing episodes lacked an observed subsequent parity because follow-up ended in June. Accordingly, recovery probabilities should be interpreted as conditional on continued observation to the next consecutive parity. A formal competing-risks analysis distinguishing reproductive recovery from specific removal outcomes would require complete information on the timing and causes of herd exit.

Finally, the reproductive-stage analysis should be regarded as exploratory. Although the lower recovery probability following repeated underperformance in later-parity sows is potentially important, the overall interaction between reproductive stage and performance history was not statistically significant. The relatively small number of episodes involving prolonged underperformance, particularly three or more consecutive observed low-performing parities, also limited precision for some stage-specific comparisons. Validation using independent multi-herd populations with larger numbers of persistently underperforming females will be required before stage-specific recovery probabilities can be incorporated into formal retention recommendations.

## 5 Conclusion

Reproductive underperformance in commercial sows was more often transient than persistent, with most low-performing episodes followed by recovery at the subsequent parity. However, the probability of recovery declined progressively with repeated underperformance, indicating that reproductive history provides more meaningful information for sow retention decisions than a single poor reproductive cycle. Reproductive stage may further inform this interpretation, although evidence for stage-dependent differences was not conclusive. These findings support the use of longitudinal reproductive records to distinguish potentially temporary from persistent underperformance rather than relying on single-parity thresholds for replacement decisions. Multi-herd validation incorporating health, management, and economic factors is required before the approach can be translated into formal retention guidelines.

## Data Availability

The original contributions presented in the study are included in the article/Supplementary material, further inquiries can be directed to the corresponding author.
