# Supplementary material for: Persistence and recovery of reproductive underperformance across successive parities in commercial sows
Source: Front Vet Sci. 2026 Oct 5;13:1980366. doi: 10.3389/fvets.2026.1980366 (PMC13678170; doi:10.3389/fvets.2026.1980366)
Supplement: Supplementary file 1 [file Table_1.docx]

Table S1. Type III tests of fixed effects from linear mixed-effects models for reproductive performance traits.

| Response | Breed | Parity_group | Season | Year |
| --- | --- | --- | --- | --- |
| Total born | 64.83 (<0.001) | 114.83 (<0.001) | 2.26 (0.080) | 17.33 (<0.001) |
| Born alive | 88.46 (<0.001) | 83.48 (<0.001) | 4.00 (0.007) | 19.32 (<0.001) |
| Piglets available before weaning | 97.39 (<0.001) | 55.81 (<0.001) | 4.84 (0.002) | 20.31 (<0.001) |
| Piglet weaned | 97.40 (<0.001) | 55.15 (<0.001) | 5.04 (0.002) | 20.19 (<0.001) |
| Gestation length | 15.45 (<0.001) | 7.76 (<0.001) | 26.67 (<0.001) | 110.75 (<0.001) |
| Lactation length | 437.93 (<0.001) | 39.30 (<0.001) | 12.09 (<0.001) | 51.37 (<0.001) |

Table S2. Adjusted least-squares means (± SE) of reproductive performance traits according to breed, parity group, season and year

| Factor | Level | Total born | Born alive | Piglets available before weaning | Piglets weaned | Gestation length | Lactation length |
| --- | --- | --- | --- | --- | --- | --- | --- |
| Breed | Camborough | 15.0 ± 0.04^a^ | 13.9 ± 0.04^a^ | 12.8 ± 0.04^a^ | 11.4 ± 0.03^a^ | 115.78±0.02^b^ | 25.22±0.03^b^ |
|  | PIC Line | 14.3 ± 0.08^b^ | 13.1 ± 0.08^b^ | 12.0 ± 0.08^b^ | 10.6 ± 0.07^b^ | 115.98±0.04^a^ | 26.40±0.05^a^ |
| Parity | 1 | 13.8±0.06^a^ | 13.0±0.06^a^ | 12.2±0.06^a^ | 10.9±0.05^a^ | 115.9±0.03^bc^ | 25.14±0.04^a^ |
|  | 2 | 13.6±0.07^a^ | 12.8±0.06^a^ | 11.9±0.07^b^ | 10.6±0.06^b^ | 115.8±0.03^ab^ | 25.92±0.05^b^ |
|  | 3 | 15.1±0.07^b^ | 14.1±0.07^b^ | 13.1±0.08^c^ | 11.6±0.07^c^ | 115.7±0.03^a^ | 25.97±0.06^b^ |
|  | 4 | 15.3±0.08^b^ | 14.2±0.07^b^ | 13.1±0.08^c^ | 11.6±0.07^c^ | 115.8±0.04^b^ | 26.00±0.06^b^ |
|  | 5 | 15.3±0.09^b^ | 14.1±0.08^b^ | 12.9±0.08^c^ | 11.5±0.08^c^ | 115.9±0.03^bc^ | 25.94±0.07^b^ |
|  | 6 | 14.9±0.09^c^ | 13.6±0.08^c^ | 12.4±0.09^a^ | 11.0±0.08^a^ | 116.0±0.04^c^ | 25.89±0.07^b^ |
|  | 7 | 14.7±0.10^cd^ | 13.4±0.09^cd^ | 12.0±0.11^a^ | 10.7±0.09^b^ | 115.9±0.04^bc^ | 25.75±0.08^b^ |
|  | ≥8 | 14.3±0.10^e^ | 12.9±0.10^e^ | 11.5±0.11^b^ | 10.2±0.09^d^ | 115.9±0.05^bc^ | 25.85±0.08^b^ |
| Season | Autumn | 14.5±0.06^a^ | 13.4±0.06^a^ | 12.2±0.06a | 10.9±0.05^a^ | 115.8±0.03^a^ | 25.93±0.04^a^ |
|  | Spring | 14.7±0.06^b^ | 13.6±0.06^b^ | 12.5±0.07^b^ | 11.1±0.06^b^ | 115.9±0.03^b^ | 25.87±0.04^a^ |
|  | Summer | 14.6±0.06^b^ | 13.5±0.06^b^ | 12.4±0.06^b^ | 11.0±0.05^b^ | 115.8±0.03^a^ | 25.83±0.05^a^ |
|  | Winter | 14.6±0.06^b^ | 13.5±0.06^b^ | 12.4±0.07^b^ | 11.0±0.06^b^ | 116.0±0.03^b^ | 25.60±0.05^b^ |
| Year | 2020 | 14.6±0.07^a^ | 13.5±0.07^a^ | 12.4±0.07^a^ | 11.0±0.07^a^ | 115.2±0.03^a^ | 26.16±0.05^a^ |
|  | 2021 | 14.3±0.07^b^ | 13.1±0.07^b^ | 12.0±0.08^b^ | 10.6±0.07^b^ | 115.5±0.04^a^ | 25.21±0.06^b^ |
|  | 2022 | 14.4±0.07^ab^ | 13.2±0.07^b^ | 12.1±0.08^b^ | 10.7±0.07^b^ | 115.7±0.04^a^ | 26.08±0.06^a^ |
|  | 2023 | 14.3±0.07^b^ | 13.2±0.07^ab^ | 12.1±0.08^b^ | 10.8±0.06^ab^ | 115.9±0.04^a^ | 25.72±0.06^b^ |
|  | 2024 | 14.8±0.07^e^ | 13.8±0.07^c^ | 12.3±0.08^ab^ | 10.9±0.07^ab^ | 116.2±0.04^b^ | 26.62±0.06^a^ |
|  | 2025 | 14.9±0.07^c^ | 14.1±0.10^d^ | 12.7±0.08^c^ | 11.3±0.07^c^ | 116.6±0.05^b^ | 25.62±0.05^b^ |
|  | 2026 | 15.1±0.10^d^ | 14.1±0.10^d^ | 13.1±0.11^d^ | 11.7±0.09^d^ | 116.6±0.05^b^ | 25.61±0.09^b^ |

Table S3: Sensitivity of the association between performance history and subsequent recovery to alternative piglets-weaned thresholds

| Threshold | Eligible episodes | Recovery, n (%) | History category | Adjusted OR (95% CI) | P-value |
| --- | --- | --- | --- | --- | --- |
| ≤9 | 1,899 | 1,375 (72.4) | Two consecutive vs first | 0.61 (0.43–0.86) | .004 |
|  |  |  | ≥3 consecutive vs first | 0.29 (0.13–0.65) | .003 |
| ≤10 (primary) | 2,948 | 1,839 (62.4) | Two consecutive vs first | 0.73 (0.58–0.91) | .005 |
|  |  |  | ≥3 consecutive vs first | 0.55 (0.36–0.85) | .007 |
| ≤11 | 4,240 | 2,239 (52.8) | Two consecutive vs first | 0.73 (0.62–0.86) | <.001 |
|  |  |  | ≥3 consecutive vs first | 0.57 (0.44–0.74) | <.001 |

For each threshold, performance-history categories and recovery status were reconstructed independently. Recovery was defined as piglets weaned above the corresponding threshold at the immediately subsequent consecutive parity. Models were adjusted for current parity, maternal genetic line, current-parity piglets weaned, prenatal loss, gestation length, lactation length, year and season, with robust standard errors clustered by sow. First low parity was the reference performance-history category.

Table S4: Magnitude of change in piglets weaned and adjusted subsequent performance according to low-performance history

| Performance history | n | Current PW, mean ± SD | Subsequent PW, mean± SD | Change in PW, mean ± SD | Median change (IQR) | Improved, n (%) | No change, n (%) | Declined, n (%) | Adjusted β (95% CI) | P-value |
| --- | --- | --- | --- | --- | --- | --- | --- | --- | --- | --- |
| First low parity | 2,407 | 8.37 ± 1.88 | 11.00 ± 2.99 | +2.64 ± 3.51 | +3 (1 to 5) | 1,822 (75.7) | 188 (7.8) | 397 (16.5) | Reference | — |
| Two consecutive low parities | 445 | 8.36 ± 1.91 | 10.70 ± 3.09 | +2.32 ± 3.52 | +2 (0 to 4) | 326 (73.3) | 34 (7.6) | 85 (19.1) | −0.52 (−0.84 to −0.21) | .001 |
| ≥3 consecutive low parities | 96 | 8.40 ± 1.83 | 9.65 ± 3.11 | +1.25 ± 3.62 | +1 (−1 to 3) | 58 (60.4) | 9 (9.4) | 29 (30.2) | −1.30 (−1.95 to −0.66) | <.001 |

PW, piglets weaned; IQR, interquartile range. Change in PW was calculated as subsequent-parity PW minus current-parity PW. Improvement, no change and decline denote positive, zero and negative change, respectively. Adjusted β coefficients are from a Gaussian linear regression model of subsequent-parity PW, with first low parity as the reference category. The model adjusted for current-parity PW, parity, maternal genetic line, prenatal loss, gestation length, lactation length, year and season, with robust standard errors clustered by sow
